# Anterior Uveitis as an Initial Manifestation of Polymyalgia Rheumatica

**DOI:** 10.1155/2011/621241

**Published:** 2011-05-10

**Authors:** Hiromasa Tsuda, Kozue Tanaka, Shuji Kishida

**Affiliations:** Department of Neurology, Tokyo Metropolitan Cancer and Infectious Diseases Center Komagome Hospital, 3-18-22, Honkomagome, Bunkyo-ku, Tokyo 113-8677, Japan

## Abstract

A 74-year-old woman without contributory medical history presented with acute iridocyclitis in the right eye. Although the iridocyclitis disappeared within two weeks under topical steroid, she complained of acute progressing bilateral shoulder pain and morning stiffness of upper extremities. She was diagnosed as having polymyalgia rheumatica (PMR), and iridocyclitis was considered as its related manifestation. PMR and giant cell arteritis (GCA) are closely related conditions and frequently occur together. GCA with uveitis has been rarely noted. However, ocular symptoms in PMR have not been previously mentioned. This is a first reported case of PMR presented with uveitis, without a complication of GCA. This anterior uveitis might be caused by ischemia of the posterior ciliary arteries and their branches.

## 1. Introduction

Polymyalgia rheumatica (PMR) is inflammatory condition of unknown cause and characterized by aching and morning stiffness in the cervical region, shoulder, and pelvic girdles [1–3]. On the other hand, in giant cell arteritis (GCA), a chronic vasculitis of large and medium-sized vessels may develop. Both diseases are closely related conditions that affect persons of middle age and older and frequently occur together [2, 3]. Therefore, these may be considered as different phases of the same disease [2, 3]. In GCA, ischemic visual manifestations are commonly observed [2–4], and uveitis as its complication has been rarely reported [5–9]. However, the relevance of ocular manifestation and PMR has not been noted. Here, we describe a first reported case of PMR with acute anterior uveitis as its preceding manifestation, though GCA was not complicated.

## 2. Case Report

A 74-year-old Japanese woman without contributory medical history complained of gradual blurred vision in the right eye and consulted the ophthalmologist on September 2009. Her corrected visual acuities were 0.4 in the right eye and 1.0 in the left. Light and near reflexes were brisk in both eyes. There was no relative afferent papillary defect. Fundoscopic findings demonstrated iridocyclitis in the right eye and no abnormalities in the left eye, respectively. Ocular movements were not restricted. Two weeks later, her iridocyclitis disappeared and visual acuity normalized in the right eye with 4 times/day of topical 0.1% fluorometholone. However, she complained of duration longer than 1 hour of morning stiffness in the cervical region, arms, and shoulders bilaterally. As these symptoms gradually worsened for a week, she was admitted to our hospital in October 2009. In general examination, bilateral upper shoulder pain and bilateral upper arm tenderness were observed. Moreover, there was morning stiffness in the cervical region, arms, and shoulders bilaterally with duration longer than 3 hours, without motor weakness. Neither arthritis nor rheumatoid nodules were observed. There was no genital ulcer. Complete blood cell counts demonstrated mild hypochromic microcytic anemia and elevated platelet count at 48.3 × 10^4^/*μ*L. An erythrocyte sedimentation rate (ESR) of 44 mm in the first hour was demonstrated. Blood examination demonstrated an elevated level of C*-*reactive protein at 7.4 mg/dL. However, CK and angiotensin-converting enzyme level in the serum was within normal ranges. Neither antinuclear antibody titer nor rheumatoid factor was within positive ranges. Chest X-ray examination did not demonstrate any abnormalities. 6 of 7 of Bird's criteria was fulfilled for PMR [1]. because of shoulder pain and stiffness bilaterally, an onset of illness within 2-week duration, initial ESR higher than 40 mm/hour, morning stiffness duration longer than 1 hour, onset of age older than 65, and upper arm tenderness bilaterally. Moreover, rheumatoid arthritis and ankylosing spondylitis were denied, based on her clinical course as well as physical examination. On the other hand, she did not complain of headache. Neither tenderness to palpation nor decreased pulsation was observed in the bilateral temporal arteries. Thereby, biopsy of the temporal artery was not performed. As a result, she was diagnosed as having PMR without a complication of GCA. Then, oral prednisolone 12.5 mg/day was initiated. Then, her clinical symptoms markedly improved, and C*-*reactive protein in the serum and ESR normalized within a week. One month later from the initiation of oral steroid therapy, complete blood cell counts improved to within normal ranges. Thereafter, under oral prednisolone 5 mg/day therapy, neither PMR nor anterior uveitis recurred. 

## 3. Discussion

PMR is characterized by severe bilateral pain and aching involving the neck, the shoulder, and pelvic girdles, associated with morning stiffness [1–3]. The inflammatory involvement of proximal articular and extraarticular synovial structures is responsible for its typical symptoms [1–3]. On the other hand, GCA is less common than PMR [2, 3]. Vasculitis that involves large and middle-sized blood vessels with a predisposition for cranial arteries usually develops, and ischemic visual manifestations are the most feared in GCA [2, 3]. In addition, thrombocytosis is considered as a risk factor of ischemic event in GCA patients [10–12]. On the other hand, GCA patients often present with clinical manifestation of PMR showing an incidence of 40–60% [3]. Furthermore, both GCA and PMR conditions are characterized by late age at disease onset, are more common in woman, exhibit evidence of systemic inflammatory response, and generally respond well to steroids [2, 3].

There has been no reported case of PMR presented with uveitis, without complication of GCA. However, only 4 cases of uveitis due to GCA have been reported [5–8]. Among them [5–8], Bandini et al. [5] noted a case of unilateral anterior uveitis occurring 3 weeks before GCA diagnosis was made based on the temporal artery biopsy. Dasgupta et al. [6] reported a case of unilateral anterior and posterior uveitis occurring as presenting feature of GCA. Rajesh and Cole [7] wrote a case of bilateral panuveitis one year before the diagnosis of GCA was made based on temporal artery biopsy finding. Coppeto et al. [8] described a case of bilateral iritis being related to the presence of GCA. Only this case had a past medical history of PMR [8]. In the previous reports [5–8], uveitis disappeared under only topical [5, 6] or systemic and topical [7, 8] steroid therapy. In addition, Védrine et al. [9] noted that uveitis was an unusual common presenting feature of GCA and anterior uveitis might be caused by ischemia of the posterior ciliary arteries and their branches. On the other hand, positron emission tomography examination suggests that PMR without complication of GCA is a form of vasculitis [13, 14]. Moreover, even in PMR without GCA, thrombocytosis may be a risk factor of ocular or cerebral ischemic events [15]. In our patient, the anterior uveitis might have developed as a preceding clinical manifestation of PMR and disappeared under topical steroid. Furthermore, thrombocytosis was initially observed and gradually normalized under oral steroid therapy. From these results, we considered that her anterior uveitis might be caused by ischemia of the posterior ciliary arteries and their branches, which was associated with vasculitis and/or thrombocytosis due to a preceding disorder in PMR.
